# Rebalancing commercial and public interests in prioritizing biomedical, social and environmental aspects of health through defining and managing conflicts of interest

**DOI:** 10.3389/fmed.2023.1247258

**Published:** 2023-09-22

**Authors:** Barbara K. Redman

**Affiliations:** Division of Medical Ethics, Grossman School of Medicine, New York University, New York, NY, United States

**Keywords:** conflict of interest, commercial interests, public interests, environmental determinants of health, social determinants of health

## Abstract

Abstract Biomedical research is intended to benefit human beings and their health. Toward that end, scientific norms involve examining and criticizing the work of others and prioritizing questions that should be studied. Yet, in areas of health research where industry is active, it has often utilized well-honed strategies aimed at evading scientific standards and at dominating the research agenda, largely through its financial support and lack of transparency of its research practices. These tactics have now been documented to uniformly support industry products. Commercial entities are aided in this pursuit by public policy that has significantly embedded commercial interests and agendas into federal research funding and infrastructure. Therefore, to understand the resulting landscape and its effect on priority in health research agendas, traditional definitions of individual conflicts of interest (COI) and the less well developed institutional COI must be supplemented by a new construct of structural COI, largely operating as intellectual monopolies, in support of industry. These arrangements often result in financial and reputational resources that assure dominance of commercial priorities in research agendas, crowding out any other interests and ignoring justified returns to the public from investment of its tax dollars. There is no sustained attention to mechanisms by which public interests can be heard, normative issues raised, and then balanced with commercial interests which are transparently reported. Focus on research supporting approval of commercial products ignores social and environmental determinants of health. Commercial bias can invalidate regulatory research protections through obscuring valid risk–benefit ratios considered by IRBs.

## 1. Introduction

Health research agendas have frequently fallen in line with incentives in the current political/economic environment that favor commercial products and the profit they yield. This prioritization has moved unimpeded. This may have occurred in part because a solid methodology and tradition of requiring balancing of commercial products and their profit with practices/products that serve public health and the public good but which do not yield economic profit has not been established. In particular, prioritizing research that supports social and environmental aspects of health and research is necessary for biomedicine to effectively meet its commitments. Currently, uncontrolled and many times undefined commercial conflicts of interest have often overwhelmed practices/policy aimed at public interest.

Under a situation of commercial prioritization and undefined/unmanaged conflicts of interest supporting it, there is much in the professional traditions and responsibilities of medicine and of research oversight that are at stake. Research practices of commercial biomedical interests, which have managed to unwaveringly support their products, are said to have rendered evidence-based medicine an illusion (1). Under these conditions, institutional review boards (IRBs) can be hampered in their responsibility to appropriately estimate risks and benefits of proposed research, a central focus of their responsibility to protect research participants, and regulatory bodies may approve initial research plans without followup analysis and reporting of research findings in published findings. Allegations of research misconduct can be and have been made to stop/derail a program of research, specifically because it undermines commercial interests (2). Request for comment of draft federal regulations can be flooded with industry-friendly comments including from patient groups funded by commercial interests, an undisclosed conflict of interest (COI) (3). Textbooks usually do not require conflict of interest disclosure; yet, two-thirds of authors/editors of psychopharmacology texts record personal payments from one or more pharmaceutical companies (3). Seventy-two percent of professional association board members for the ten costliest diseases were found to have financial ties to industry (4). The World Health Organization (WHO) felt compelled to advise governments how to protect public health nutrition policies from commercial interests (5).

All of these examples reflect the current environment of unconstrained conflicts of interest, deliberately networked into policies/practices that might restrain commercial priorities. Among other deleterious effects, COI distorts the agenda for thoughtful prioritization of determinants of health that would significantly improve population health, with better investment of resources. Here we focus on the potential for environmental determinants of health (EDOH) and social determinants of health (SDOH) to contribute to these goals.

The question addressed in this paper is: what common set of conflict of interest standards/norms/practices is necessary to rebalance commercial and public interests in prioritizing biomedical, social and environmental aspects of health? We first consider the definition of COI, its effects and how the current situation has evolved and plays out in environmental determinants of health (EDOH) and social determinants of health (SDOH). Risks both to democracy and to science are involved. We then consider how public and commercial research interests might be rebalanced, in part by recognition and control of COI. Unrecognized and uncontrolled COI destroy trust, as do other related constructs such as complicity and corruption. This paper is largely focused on the US regulatory structure; further work will be necessary to test its conclusions in other countries and globally. Many of the examples focus on the pharmaceutical/medical device and food industries; application to other industries remains to be examined.

This paper is written in the mode of a narrative critical review, based on publications retrieved from a variety of databases (Web of Science, Scopus, PubMed) by means of the search terms noted as key words in the title page and a snowball technique searching references. Examples (which are non-exhaustive) yielded from these searches were aimed at answering the question described above. Greenhalgh and colleagues (6) note that narrative critical reviews such as this provide interpretation and critique, clarification and insight – their key contributions being deepening understanding. Such an approach is useful when considering new emerging constructs for which there is little normative or empirical literature.

Assessment of policy options and implications require development of relevant constructs and their root causes, attention to risks to democracy and to science and most especially to EDOH and SDOH. Sections 2–4 develop this content.

## 2. Conflicts of interest distort the scientific record and affect prioritization

Conflict of interest, defined as “a set of conditions in which professional judgment concerning a primary interest…tends to be unduly influenced by a secondary interest…” [(7), p. 290], has largely been applied to individuals, less to institutions and not at all at structural levels. An institutional conflict of interest (ICOI) occurs when that institution’s financial interests or those of its senior officials pose risks to the integrity of the institution’s primary interests and missions. Monetary, social and moral incentives are distinctively important in analyzing COI (8).

IRBs are arguably the most prominent bodies devoted to research regulation. Individual IRB members with a COI cannot review protocols with which they are conflicted, and independent IRB members cannot hold equity in a company whose protocol is being reviewed. Commercial IRBs would have their own policies. Yet, a recent study notes that there are no requirements under the Common Rule or Food and Drug Administration (FDA) regulations for IRBs to manage organizational COI that may come up in their reviews (9), even though IRB reviews can advantage or disadvantage the institution that employs most of the reviewers.

While institutional COIs are fairly well defined, COI is in fact heavily structural, built into relationships of the whole sector of institutions that produce and provide health care and disseminate its research. These institutions – academia and health care – are historically and normatively nonprofit but are currently required or heavily incentivized to incorporate the sector of commercial market-driven institutions and logics, which can be seen as a secondary interest. These merged relationships have been implicitly accepted as normative even though they frequently prioritize the secondary interests which can overwhelm primary social missions of academic and institutions. A structural conflict of interest might be defined as a set of conditions in which the primary interest of a sector of institutions is unduly influenced by the interests of another sector of institutions with different and often conflicting values.

A long list of industries (including tobacco, chemical, pharmaceutical, and food) have used the same power structure and playbook as well as their large-scale role in financing and designing the majority of research, to set the research agenda and to normalize corporate influence over it. COI is widespread, in part because these industries have purposefully infiltrated multiple networks in order to assure their commercial interests, a documented pattern that has only recently been called out as violating the primary interest of health care/research (10).

Compromising effects of COI occur in many parts of the scientific record not only in individual studies but also in production of systematic reviews, which inform policy. For example, Zhou and Xie note that industry sponsorship bias (from COI) is significant in cost effectiveness analysis for oncology, which is used to inform treatment coverage and pricing policy (11). Industry-funded cost effectiveness analyses were significantly more likely to report effectiveness results in favor of the new treatment than were studies without industry sponsorship, a consistent finding across research areas (12).

In a further example, a cascade of food scandals in the United Kingdom (UK) resulting in loss of public trust is thought to have involved capture of regulatory institutions by the industry. The example suggests that to avoid such capture, boards and advisory committees should not include anyone with COIs, and companies should not design or conduct safety studies (13). Likewise, a United States (US) senator called for a probe of COI on the federal panel overseeing dietary guidelines; several prior guidelines requiring disclosure were apparently not followed (14). In yet other examples, a review of robot-assisted anti-reflux surgery found multiple violations of good research practices including not providing statements about COI (15). And in a study of robot-assisted vs. laparoscopic cholecystectomies, authors of robot positive studies received higher amounts of industry payments on average (16), a common pattern in many interventions.

A less obvious incarnation of conflicted interests at a structural level can be seen in establishment of translational research centers, funded with significant public monies. Built on the notion that government, academia and industry must come together to more rapidly move research into products, translational science does not deal with or assumes away any COI that may exist among these parties. The Bayh-Dole Act of 1980 laid the legislative foundation for translational science, some note, as a way to prioritize industry needs and to embed private interests in the infrastructure of biomedical research (17) by supporting initial product development with public money.

In addition to operating at three levels (individual, institutional and structural), management of COI is incompletely theorized. By itself COI simply points to existence of links within these networks but does not provide information about whether and/or the degree to which the primary interest has been compromised. This means that current COI disclosure requirements, if and when they exist and are followed, provide little information about the level of risk to the primary interest/research integrity.

In summary, individual COI are most commonly addressed, institutional COI less so; neither is adequately managed by recusal from the conflicted activity. But structural COIs yield the greatest impact, yet are not commonly identified or addressed. They are hidden by acceptance of market ideology as normative including being inserted into federal research funding programs which lack acknowledgement of them, especially at the structural level. Why is distortion of the research agenda favoring commercial interests allowed and where can its roots/structural causes be located?

### 2.1. Why is commercial distortion happening?

Commercial distortion is happening because it is not only allowed but encouraged, consistent with widely accepted ideologies/policies and incentive structures.

Various versions of short term capitalism, embedded in the health care system, clearly conflict with core values and responsibilities of health care professionals and researchers and with fair support of their commitment and contribution to the common good. Current practices also result in injustice and lack of fair compensation to parties contributing to research production. Several examples are illustrative. First, intellectual monopoly capitalism has been well described for the pharmaceutical industry, which largely outsources research and development to multiple innovation networks including research universities, keeping to themselves the knowledge produced and sole access to it. In general, producers of that knowledge do not receive fair compensation, and the monopoly uses its advantageous position to steer the public and academic research agenda toward their priority areas. Under this arrangement, pharma outsources risks of early research and monetizes it for its own benefit (18).

A second example of distortion in favor of commercial entities may be found in the prominent rationale for public financial support of medical product development: that the rate of discovery for life-saving treatments has decreased over time while costs have increased. Why is public subsidization the necessary solution? One consideration is that financing of drug development depends on external sources of money, exposing companies to aggregate market risk. Those treatments valuable for society may not attract sufficient private capital to support their development. This leads to the explanation that in such cases, public-private partnerships including government guarantees, are required (19). Surely, such a financing model might be rebalanced so that the public receives a fair return for its investment, which would require substantial renegotiation, for example, for lower drug prices.

Third, a series of reviews published in The Lancet note that products/practices of some companies (think tobacco, some chemicals) cause significant harm which under current arrangements is externalized to (paid for by) the public. Not only do company taxes not begin to address these costs, health systems cannot cope with the burden of disease those products are causing, draining funds from needs such as housing and equitable health care. Current norms are not inevitable; commercial entities will need to meet the true costs of the harm they cause (20). Some suggest that contemporary capitalism needs to increase its compatibility with health (21). This assertion will be addressed in a separate paper.

A root cause of these and other such arrangements is that twenty-first century law, politics and regulation reward monopoly business practices (22); this explains the strong role of markets in structuring contemporary medicine. Monopoly capitalism will not address social and environmental aspects of health, even though they are more influential for health than is health care itself. It ignores not only research necessary to support the public health agenda but also the voice of the people in a democracy to set a research agenda, which is largely an investment of public resources.

Can the construct “conflict of interest” capture these structural commercial advantages, which have the effect of assuring that corporate agendas promoting their products are dominant, not only over commercial responsibility to the public good, but also over non-commercial aspects of public health? Even at individual and institutional levels, COI is undertheorized, making it difficult for individuals (and institutions) to identify situations which involve COI, much less manage them. But the examples of structural COI provided above are not considered to fit the definition of a primary interest (production of health) being unduly influenced by a secondary interest (commercial profit); therefore, their harms are not acknowledged or considered necessary to control.

## 3. Risks to democracy and to science from unchallenged commercial dominance

Risks of current commercial dominance and lack of control of its COIs reach deeply into our governance commitments and affect science, its practice and regulation.

Democracy requires a standard for validity of evidence, institutions that certify it and public involvement in deciding when that evidence is ready for application. Indeed, Fukuyama suggests that democracies cannot survive if they are unable to establish a hierarchy of factual truths (23). Likewise, states are necessary to oversee markets and to provide public goods that markets by themselves will not provide, and there is no reason why economic efficiency needs to trump all other social values (23). But some note that the governing ideology in America has become cutthroat, supporting the notion that those who have not been successful just did not work hard enough and therefore do not deserve help. This view has been used to justify lack of attention to the social and environmental determinants of health.

Corporations dominate our economy and shape our democracy, and millions of Americans are subject to these incentives/pressures in their daily work lives. The harm they are causing must be challenged through evidence-gathering, lawsuits, media attention, political movements and new laws. In response, industry uses its power to block policies that threaten its interests, redirects attention to “other problems,” discredits challenging their practices, and disputes the facts and delays decision making until the issue fades.

Magic of the marketplace and down with Big Government was a mantra promoted by Big Business during the twentieth century (24). The form of capitalism we choose should encompass a view of where markets are successful and where unsuccessful, understanding that they need to be managed and subject to regulation. Governments are necessary to provide public goods and to address social costs of business but can, under market essentialism, be left in a weakened position to fulfill these functions. If properly balanced, commercial entities and government should play complementary roles (24, 25). It is again important to note that under an ideology of market essentialism, a notion of conflict of interest does not exist.

In summary, the role of markets in a just society should be structured to meet democratic goals, and a democratic society should think carefully about where and how to use markets. Also of note, we have allowed commercial biomedical markets to operate with extensive hidden conflicts of interest, depriving the public and the scientific community of an appropriate role in setting research agendas. What are the most direct effects on the practice of science and on its regulation?

### 3.1. Effects on science, its practice and regulation

In support of market essentialism, commercial science has been allowed to operate opaquely, with no democratic accountability. Alternatively, academic science directly supported by public money is subject to regulation consistent with democratic values although incompletely implemented.

We are left with two irreconcilable standards for scientific practice and knowledge – those expected of academic science and those for commercial science. The latter can decide what counts as relevant evidence, select research design and outcomes, control evidence and interpret it, often to their advantage. Any element of commercial science including clinical trial protocols, quality control procedures, safety and efficacy data are protected as trade secrets. According to an analysis by Feldman (p. 40), (26) FDA releases only a summary review of “pertinent” studies, not the complete set of evidence that substantiates its decisions. Trade secret protections inhibit outside auditors from reviewing clinical study data and findings, even though latent conflict of interest is present in all commercial trials. The U S Supreme Court has supported confidential commercial information as a broad category, inhibiting public access to this information through Freedom of Information Act (FOIA). (26). In a democratic society, no institution should be allowed to govern itself; yet, commercial entities and the research and products they produce are largely self-regulated.

While federal agencies have largely acceded to corporate claims of trade secrets, they do have statutory and constitutional authority to obtain and divulge otherwise secret information when doing so serves the public interest. But despite law establishing the ClinicalTrials.gov database, National Institutes of Health (NIH) and FDA have not enforced the law’s reporting requirements (27), implicitly acknowledging that commercial research practices of nondisclosure and lack of public access to trial information are acceptable.

To see the chasm between academic and commercial science, consider the notion of Open Science, touted as a public good, because freely open and shared data can support more rigorous research findings. Currently, through their hold on Big Data, commercial entities have full access to free data sources including public data. Capps argues that commercial sources should be excluded from data commons unless operating transparently, with fair contribution of their own data to the commons and fair compensation to the commons. Instead, there is a blurring of capitalist and public health agendas, which allows commercial access to data based on the legitimacy of surveilling people’s health (28), a public health function from which commercial entities should be barred. Lacking such an equitable arrangement, Open Science is being abused. Ethical concerns go well beyond privacy, sectors – open science practices are meant to serve the public good (primary interest) but can be usurped by nontransparent and noncontributory commercial interests.

From another perspective and in light of expanding notions of COI, current practice in public funding of science should be examined for its own conflicts of interest. A scientific establishment is largely in charge of how and to whom money flows, with public funders largely being supportive of the recommended allocation. But disciplinary specialists’ interest is to extend resources beneficial to them, not necessarily to the public, undermining the sense that science, under this funding system, is surely a public good. “Peer review routinely conflates judgments about the validity of work judged on its own terms and in terms of some larger disciplined-based agenda which, in the end, may matter only to other academics” [(29), p. 31]. Scientific autonomy could be seen as self-certifying academics entitled to monopoly ownership over science, largely removed from societal concerns (29). This arrangement also is a COI, although not currently perceived as such. Research for the public good (the primary interest) can be diverted for personal ends of scientists and/or their disciplines (a secondary interest).

The current imbalance favoring commercial control of science undermines both mechanisms of societal governance and the responsibility of science to produce public good. It is against this backdrop that we examine how COIs play out in EDOH and SDOH, both essential to the production of health but generally lacking commercial attention and viability.

## 4. How do COI play out in environmental and social determinants of health

Both environmental determinants of health (EDOH) and social determinants of health (SDOH) are seen not only as commercially nonviable but also as interfering with production of commercial products and as objecting to additional harm those products may inflict on the environment or on individuals through social institutions that serve them.

### 4.1. Environmental health environmental determinants of health (EDOH)

A substantial proportion of disease risks for common complex diseases is attributable to environmental exposures and pollutants. The exposome is defined as the cumulative measure of environmental influence over the lifespan, and is known to induce biological responses in every layer of human biology, translating into substantial disease risk (30). The central question of COI in environmental health is a justifiable balance between protection of the environment and its effects on human health, and commercial interests which often impact the environment for profit.

As has been noted above, commercial entities dominate the research agenda by: information strategies, constituency building, financial incentives largely supported by governments and by legal and regulatory practices that protect the total opaqueness of their data/findings. States nurture partnerships with industry to create high growth and expand sectors of their economies (31). Industry also uses “corporate social responsibility” programs to deflect attention from their commercial goals, even though such programs have not shown significant evidence of positive environmental impact. The true purpose of these programs is marketing, production of good will and staving off governmental regulation. Such “successes” are possible to allege in the presence of lax regulation with little oversight (32). What practices cross the line to damage the environment and create harmful health effects? COI examples in environmental science and its health effects are illuminating. They may be structural or practices that introduce bias favoring commercial views.

Structural conditions in U S environmental policy assure an imbalance, favoring business interests. Federal environmental regulations weigh, through cost/benefit analysis (CBA), protection of human health and environment against cost to existing business interests of complying with those regulations (CBA). CBA justifies health harms and even death when it is judged to be too costly to avoid them, largely precluding primary prevention (33).

A prime example of COI in the real world is a case study [reported by Rajao and colleagues; (34)] of Brazilian environmental policies, particularly on issues of climate change and deforestation, where fake scientific controversies have influenced policy which would be contrary to commercial interests. A small group of Brazilian scientists seriously impacted such policy by manufacturing uncertainty and disregarding scientific literature. They allegedly manufactured “pseudo-facts,” an affirmation at odds with the established literature but which aimed to appear as scientific facts. Such efforts are often supported by sectors in the economy (in this case, agribusiness) interested in delaying policy. Although disagreements among researchers are part of science (genuine scientific controversies), some controversies are manufactured to create public perception that there is no consensus regarding a specific policy. While the scientific community is often not well prepared to deal with these fake controversies, it must vigorously rebut them to sustain its reputation as an unbiased community (34).

In another example of bias, funding in the environmental domain continues to prioritize the bio-geochemical research agenda (to support authority, interests and careers of scientists in those fields) over a robust socio-political research agenda. The COI is created when the research agenda is controlled by the power of a biological influence built into the structure of the research agenda, which dominates and continues to fund its own research, no matter the needs of the field and of society (35, 36). This same pattern can be seen at the Intergovernmental Panel on Climate Change (IPCC), part of the United Nations (UN) Environmental Program. The IPPC’s mandate is to provide governments at all levels with scientific information to be used to develop climate policies, and the Panel’s reports are a key input into international climate change negotiations. Yet, tight political control on the IPCC includes marginalization of the social sciences and indigenous knowledge and experience, in favor of the natural sciences, aimed to assure the panel’s scientific authority (37). Such a bias all but assures significant neglect of SDOH in environmental policy, at the highest level of government. A dominant scientific discourse that privileges topics without evidence that other topics are just as important, should be monitored and adjusted (38).

Uncontrolled commercial practices not only affect environmental health but create unfavorable SDOH. For example, emerging research suggests that exposure to high levels of air pollution at critical points in the life course is detrimental to brain health, including cognitive decline, dementia, learning in childhood, etc. Since the places people live play a major role in air pollution, SDOH can impact brain health (39). Likewise, environment affects mental health through neurotoxic pollutants. Genetics can explain only a portion of brain or behavioral dysfunction and since mental health issues are common, it is important to pursue relevant environmental research rigorously. Environmental disasters often result in widespread mental health consequences. If properly protected/regulated, the environment also has important mental health benefits. Current environmental health policy fails to address these mental health/brain health issues (40), with immense consequences across the globe.

Climate change offers a clear example of policy biases supporting commercial interests, with disastrous effects on health. Biodiversity loss and increasing pollution are clear signs of the planetary state of emergency (41). Like other industries largely protected by economic policy, those in the fossil fuel sector, whose products play a significant role in climate change, have deflected criticism onto individuals, who “should control their carbon footprints” and have convinced lawmakers that the science is unsettled, all in a political battle to prolong their windfall profits without having to pay for the damage they are producing. Their playbook is denial, deception, distraction and delay. This pattern has prompted the observation that neoliberal market economics is fundamentally at odds with basic human rights and environmental sustainability, and that this ideology “has gotten us into this mess” [(42), p. 266]. Such a situation could be seen as a conflict of interest since institutions have a balanced responsibility including to the common good.

Such deliberate undermining of the research base for climate change confuses and delays prioritization of research agendas toward inaction and mistrust of science. Unless such widely enacted obfuscations of the scientific record are detected and called out immediately, commercial interests will continue to dominate. Such reprehensible behavior totally denies commercial responsibility to the common good, yet is not illegal. In fact, it is widely tolerated and not seen as the COI that it is.

In general, these biases have not been directly confronted. Instead, attention has been turned to new, more comprehensive frameworks that are emerging/evolving. Internationally, a One Health approach, integrating human-animal-environment interfaces, is gaining support in control of endemic and emerging infections and neglected tropical diseases. Existing legislation and global governance instruments do not adequately address the drivers of spillover and spread of emerging and endemic disease (43). But once again, food safety goals remain subordinate to trade objectives of agri-business (44).

While the One Health approach has historically focused on zoonoses, Planetary Health is a newer concept, focusing on the environment, particularly climate change and human health, and on social determinants of human health. Planetary Health refers to the health of human civilization and the state of the natural systems on which it depends. One Health and Planetary Health are highly complementary fields with solid leverage for translation into policy and practice (45).

In summary, while climate change is a prime example of domination of commercial values, it also demonstrates challenges to rebalancing to values of public good. Because of enormous economic shifts necessary to deal with climate change and environmental health, because such policy is not only national but also necessarily international, and because environmental health strongly affects biomedical and social aspects of health, the structural conflicts of interest between profit and the public good in environmental health are especially salient.

### 4.2. Social determinants of health (SDOH)

Large scale institutions that structure a society shape distribution of downstream social determinants, including those affecting health. At what point does the responsibility of societal institutions to the common good require reordering/rebalancing agendas to support health?

SDOH is an umbrella term to refer to economic, cultural and ecological determinants of health. Although clearly tied to health, social determinants have largely resided outside the structure of medical care. The literature documents the struggle, especially in inpatient settings, to document SDOH and to take action/make referrals. For example, Cordova-Ramos and colleagues found only 23% of level 2–4 neonatal intensive care units (NICUs) reported standardized SDOH screening, even though the American Academy of Pediatrics has recommended such screening since 2016 (46). SDOH disproportionally affect families with preterm infants. Although the infants remain long enough in the inpatient setting that such screening and referral could be accomplished, there are few to no incentives to do this work or to assure action. The constant question is: why not just pay for social services directly, not through the medical care system? Stated differently, to what degree should the institutions that constitute health care be responsible for producing health, setting aside other agendas such as sustaining the medical profession, scientific institutions and producing profit?

A social determinant not widely recognized but on full display in the COVID epidemic is status distrust of scientific experts. Commonly thought to reflect lack of understanding of the science, group distrust of scientific experts can be related to distance in social status.

Experts are given high status and discretion over their work which can be used to assert both their views and their status. Low status individuals believe they have less influence on collective decisions which may not reflect their values, and that their vulnerability will be taken advantage of, especially in cases of conflict of interest. Thus, the ground of status distrust is the perception that high-status individuals do not care about the fundamental values and interests of the low-status individuals, even though experts believe themselves to be well-intentioned and to be fulfilling the responsibility of their primary interest (47). And what efforts should these institutions be expected to make to ameliorate SDOH?

The COVID experience exposed profound effects of SDOH and limitations of current approaches. It also left questions about whether and how health care and other institutions have a responsibility to reach far enough upstream to address social conditions that create ill health. Emerging policy initiatives should help but have not yet been thoroughly engaged. Value-based payment (paying for outcomes) should incentivize physicians and health care systems to initiate SDOH screening activities, but also require quality measures for social risks, standardized data and implementation assistance (48). Research including cost effectiveness and impact of a value-based payment system is needed. So far, the research base does not support the common sense view that more contacts in social needs interventions would lead to better health outcomes (49).

Currently, the public benefit policy requirement for hospitals has largely been met through subsidizing uncompensated care. More recently some have addressed housing assistance (50). Population level investments have not necessarily been seen as the role of the health care system. Improving equity will require structural change and redirection of resources (51).

More basically, the SDOH approach has failed to address the roots of social determinants – the political-economic arrangements that cause the maldistribution. For example, medical debt, driven by inadequate coverage so insurers can maintain profits, undermines SDOH, causing food and housing insecurity. Vesting the commercially dominated health sector with responsibility to address SDOH effectively, is said to privatize social welfare interventions (52) – is this the responsibility of health care institutions? Current inclusion of SDOH as a diagnostic category in the International Classification of Diseases can be seen as a victory but a very partial solution. It also legitimizes individual social needs only when they give rise to poor health and ignores acting on poor health at the population level (53).

In summary, efforts to address SDOH have not fit into mainstream health care practice and reimbursement, and lack of investment in a research base signals low priority. But it is important to note that insofar as healthcare practice and policy are driven by medical assumptions, focused on individual provider-patient relationship, they will be blind to inequalities, for which a public health framework, dealing with populations, is a much better fit. Indeed, lifestyle and behavioral approaches still dominate in much chronic disease care, problematic because individuals alone are not empowered to account for or respond to all of the impacts and influences on their health (54). Addressing SDOH requires revisiting socially defined missions and primary responsibilities of health care institutions to make their responsibilities clear, subsequently determining whether their secondary interests such as a level of profit constitute a COI.

Section 5 addresses actionable recommendations. Section 6 reminds us of the broader ethical implications of poor COI management and of how a more explicit move to assurance of research integrity could help to bring COI under control.

## 5. Rebalance of public and commercial care and research interests through controlling COI

A rebalance, using COI management as a leverage point is in order. It could be addressed by: adapting to a more balanced form of capitalism; adopting a fairer way to set research agendas including management of partnerships so that COI is well controlled/managed; and requiring a common, rigorous set of research practices across all of science.

### 5.1. Re-examining contribution of current capitalist practices to a goal of public well-being

In the last 50 years domination by transnational corporations, financial markets and globalization (including de-regulation, and privatizing previously public responsibilities) have been prominent. Important medical innovations achieved in the past, should be re-examined under current conditions. Institutions involved in supporting business activities designed to generate profits and increase market share influence patterns of health, disease, injury, disability and death within and across populations. These forces have expanded, including not only the for-profit companies but also trade associations, advertising/public relations firms, lobbyists, financial institutions, and probusiness think tanks (55). Transnational corporations have consistently lobbied for policies that structure economies worldwide to their benefit, through taxation, competitive and trade protections, hence safeguarding their interests. In addition, in order to reach their goals, companies are embedded in the political, legal, social economic and cultural fabric of a country (56).

Biomedical research and the rules that guide it are performed within structural conditions imposed by capitalism and liberal and neoliberal ideologies, often dedicated to the prominence of markets and the view that individual choices should not be interfered with. Under these ideologies, policymakers tout the market as an efficient means of allocating scarce resources, including for health. Such views/practices are highly profitable for corporations but ignore needs for which markets fail, including those affected by SDOH and environmental factors, often chronic diseases (57). This current approach supports wealth generation by nations and globally. Assisted by huge lobbying and campaign contributions (58), commercial entities have established themselves as key stakeholders regarding rules of trade and commerce and their regulation (59). Corporations have positioned themselves to have a right to participate in decision making around health, especially in product regulation. These assumptions are supported by governing ideology, not just by lobbying and campaign contributions and may be called the “political determinants of health” (60).

The COVID-19 pandemic serves as a global biomarker for neoliberalism. Much of the health care system in the US and elsewhere, which had been privatized, collapsed in the first few months of the pandemic, and neoliberal capitalism constrained the WHO’s ability to obtain protection for much of the world’s population (61).

Social movements are necessary to modify 21st century capitalism which has usurped and controlled resources including science and technology, in part through political practices, toward the goal of commercial profits rather than for public well-being. Such a diagnosis is increasingly accepted; a vision of a desired future is being built. Mechanisms by which such movements can create a more desirable future include: growing the public sector including public health, which has been greatly diminished, to compete directly with corporate entities; strengthening democracy by reversing rule changes that have benefited corporations; and making science and technology public property. Spaces free of corporate influence should be established. Institutions such as universities will need to decide whether they are willing to support academic values such as eliminating conflicts of interest, fully reporting of research results, and/or accepting industry money only through a wholly independent third party (62, 63).

Public concerns about social problems that capitalism is alleged to have created (climate change, income inequality) require substantive changes in business norms and culture, to place social missions on a more equal footing, rebalancing the twentieth century near total concern with optimizing financial returns (64). Some have alleged that organized medicine in the US was thoroughly infected with capitalistic excess including unchecked and concealed commercial influences on practice and research, constituting a serious conflict of interest on its social responsibility to optimize the public’s health (65). Its almost singular focus on acting as a protective guild, backed by economic interests can still be seen today in the separation of medical and public health interests and in lack of attention to SDOH. “Physicians averted their gaze from the social determinants of disease while rushing in to cure them when it was often too late. Their representatives did nothing about the miserly funding of public health agencies” [(64), p. vii]. This history shows a profound conflict of interest between medicine’s ethical/social role and its economic interests (65), but does not address whether this reflects the current state of affairs or how physicians function.

### 5.2. Take control of resetting health research agendas including diligence in constructing partnerships that appropriately support the common good

Several sources note that there is no global consensus on a standardized methodology for health research prioritization (66, 67) or for addressing COI effects on it. Others provide examples of serious gaps in disease investigation, especially related to EDOH and SDOH. For example, most studies of Parkinson’s Disease, assess genetic risk without consideration of environmental exposure (68). Second, the prevailing health and biomedical science research agenda is mostly focused on molecular biology and prioritizing research on pharmacological interventions over socio-environmental factors influencing disease onset or progression. Testoni and colleagues found bias in academic research agendas toward cancer (although ignores carcinogenicity from environmental pollution) and cardiovascular research – areas in which drugs are highly profitable. Research on prevention and assessment of socio-environmental factors is negligible (69).

Industry control of the biomedical research agenda is forcing social and environmental aspects aside, both financially and ideologically. Control of partnerships between commercial and public sectors can rebalance that agenda. Governments are responsible for public good; corporations are not, even though the current assumption is that inclusive multi-stakeholder coalitions are necessary to assure the public good. They are not, and instead, corporate involvement and philanthropy restrain governments from proper regulation and assure corporate agendas prevail, leading to neglect of long-term structural corrections. This situation is a clear conflict of interest and is deeply ethically problematic.

Government bodies, academic institutions and civil society organizations have responsibility to develop counter-strategies to insulate themselves from industry influence and in the process to wean themselves off industry funding or to redirect it to independent entities that can disburse it without COI. Understand that “partnership,” which may be forced on researchers and research institutions by funders, masks power differentials and requires systemic analysis that takes in account the cumulative effects of commercial interests, manifested through their webs of influence. “Partnerships” which hinder public agencies and academic institutions’ ability to meet their mission/purpose should be rejected. A norm of appropriate but rigorous separation should prevail.

More specifically, standards/norms/practices should:

- Recalibrate the boundary between commercial trade secrets and public need to know, especially through FDA release of publicly-relevant data.
- Pay not for drugs but for their therapeutic effects. This should require strict and auditable evidence-based decisions on drug approval.
- Add scientists to the Open Payments database, as nearly half of faculty in nonclinical departments have a relationship with industry, adding to the bias toward commercial research agendas (70).
- Establish a “public track” which would remain in the public domain, to fund development of novel pharmaceutical molecules (70).
- Publish full study details and data (70).
- Require a firm commitment to preventing commercial interference with public health interests, along with clear boundaries for for-profit involvement in research.

Again, more specifically, conflict of interest can be decreased at the start of a “partnership” relationship, by: giving the powerful industry lower degrees of participation, limiting its role to consultation or to simply providing information, not involving them in education and awareness, and involving them only in implementation rather than in policy formation (71). It is important to note that at an international level, WHO policy is now predicated on the concept of a fundamental COI between the tobacco industry and public health. “Partnerships” are to be rejected, although not all countries observe this policy. Notably, such a strong position has not been attained for other commercial determinants of health (CDOH) such as alcohol control (72).

### 5.3. Required extension of academic norms and regulation to commercial science to support research integrity

Under the guise of support of economic goals and competitiveness, the US has not only allowed but championed two separate systems of scientific practice and regulation (public and commercial), with studies from both sectors intermixed in the scientific record and no single standard by which both, but especially commercially based/sponsored science are available to be judged.

The move toward research integrity being reconstructed will never occur until industry research practices and areas of poor research practice are under control. Bodies of evidence will never accumulate to an actionable level, and if they do (through exclusion of research contaminated through conflict of interest), will be challenged, denigrated in an effort to stave off regulation. Corporate research interests influence science by driving research agendas; manipulating design, methods and conduct of research; selectively publishing findings or interpretations of findings; attempting to change evaluation of science especially for its use in policy. Industry initiatives are disguised as ways to promote research integrity (73).

It is important to note that industry has no concept of conflict of interest but rather sees scientific knowledge as a risk that can contribute to corporate demise. Therefore, in order to delay regulation, it uses multiple tactics well described in The Play Book to discredit science. There is no formal punishment for these activities and no consequence for nondisclosure of industry support for activities of scientific denial. Scientists prominent in the field of study that challenges industry interests are attacked, usually ill prepared to counter allegations and often unsupported by their institutions that wish to retain industry funds (another conflict of interest). Effects on research integrity and the public’s perception of it are highly detrimental (74). Concerns about need for reconstructing research integrity to improve current research practice play a role in this conversation. If quality of research were better assured with clear regulations (including self-regulation) and monitoring, industry actions as well as other problematic research practices could be exposed and challenged.

Some initiatives in support of research integrity are being normalized. Preregistration of all research studies (unless exploratory) should be required, as is now largely the case for clinical trials, and certainly mandatory for publication in the scientific literature. Preregistration involves declaring research plans (hypotheses, design and statistical analysis) in a public registry before the research outcomes are known; adherence to that plan will be monitored as discrepancies appear to be common. Preregistration joins other tools such as statistical tools to differentiate signals from noise, randomization which helps isolate causal mechanisms, placebos which help to control for participant reactivity (75). Also needed are standardized metrics for assessing exposures to commercial determinants of health (CDOHs) over place, time and population, through biological, environmental and other social pathways.

Finally, law affects health by structuring, perpetuating and mediating social determinants of health but also by improving fairness in social arrangements (although not always achieved). Public health law involves legal powers and duties to create the conditions to provide health. To date, most legal focus has been on founding and governing health institutions (health care) rather than on inequalities that determine poor health outcomes. The role of law should be on improving the broader social conditions for good health, thus forming legal determinants of health (76).

## 6. Relationship of conflict of interest with related constructs: complicity, corruption, trust

In this paper, evidence of COI has been encountered but frequently unacknowledged, ignoring the public responsibilities of commercial entities, commercial practices and agenda setting in research, as well as delegation to the scientific community to set research agendas, and neglect of EDOH and SDOH. Technological changes now support widespread availability of data with the normative concept of Open Science, without rules for fair access.

Related constructs provide further meaning to poor management of COI. Complicity means being an accomplice, a partner in wrongdoing. In the era of research impact and partnership/stakeholder engagement, Martin suggests complicity has become institutionalized. Expectation/forced collaboration with industry as a condition of funding certainly involves addressing financialization and industry activities and expectations that they will shape the health care and research agenda. Such a set of expectations clashes with researchers’ responsibility to remain independent, principled and critical. Complicity might involve lending legitimacy to industry agenda, failing to call out or question unacceptable practices, ignoring the experience of patients and marginalized groups, adopting the norms and values of the dominant actor that wants to avoid scrutiny. Direct suppression or deliberate non-engagement may occur in order to make research the servant of economic prosperity (77). Industry does not have a good record of engagement with SDOH and environmental impacts on health, consistently and across many industries, protecting their products and asserting individual choice is the only necessary protection.

“Corruption is…the public perception of the intentional hijacking of a benign or benevolent social entity (a system, organization, or institution) for the benefit of a select group who pose as fair traders on behalf of the entity. It is the intentional leverage of trust or assumption of beneficence that distinguishes corruption” [(78), p. 526]. All institutions are “corruptogenic” – deviating assets from the proclaimed functions of the institution and once underlying norms and systems have adopted corrupt ways, they are difficult to reverse. Corruption can be imposed by one system on another (see above paragraph on complicity). Establishment of rules and constant oversight with clear punishment seems the best current approach. It is important to note that decisions favoring corruption are not inevitable if governments make different decisions. Although not suggesting corruption, Berwick alleges that US health care institutions (companies, insurers, hospitals and others) are in the grip of financial self-interest (79).

Conflict of interest, complicity and corruption all damage trust, which in turn undermines cooperation. A trusting physician-patient relationship is necessary for medical practice but is viewed through the social context of institutions (80). The presence of a medical device representative, employed by the producing company and incentivized by the amount of sales, to advise the surgeon how to use the device, is largely unregulated. Surely, this situation should be seen through a real or perceived COI lens. The COI is exacerbated if the physician is involved in development of the device. A commercial party taking such a role in the provision of care is seriously problematic and should be regulated by a clear policy about appropriate roles with active oversight or alternatively, banned and a hospital employee device specialist provided (81). Trust in research communities and science institutions is also essential. This extends to fair treatment and evaluation of scientists and to how scientific communities interact with the public (82).

Perhaps the basic issue is that research accomplished with integrity has the greatest chance of meeting societal needs, and monitoring of its integrity would unmask COIs. Because research practice is – to an unknown extent - lacking in integrity, is not monitored and/or corrected, COI is allowed to flourish and its harmful effects not controlled (83).

## 7. Discussion, limitations and conclusions

Through economic ideology (neoliberalism and market essentialism) and resulting power dynamics, the research agenda has been heavily influenced by profit, way out of balance with public health/public good priorities that are essential to a functioning democracy. Privileged by law and prevailing norms, the “playbook,” especially of transnational companies whose products are or can be harmful to health, is now well understood. The battle toward a preferred balance requires the following kinds of general strategies, specifics to be **negotiated among governing bodies**, regulatory agencies, commercial interests, and the public.

- Stop commercial interference with public health/public good research and severely limit commercial involvement/roles in partnerships

- Rebalance commercial transparency and unexamined levels of information protection, as well as establishing a public track for developing pharmaceuticals
- Require evidence-based and independently audited evidence of product effectiveness at individual, population and subpopulation levels
- Recoup public investment in research products and enforceable standards for fair returns for those producing research data

It should be noted that global authorities such as WHO are moving at a glacial speed to provide governance advice for nations on commercial COI, reflecting the power of those who have benefited from the current system. Also of import is that there is no global consensus on a standardized methodology for health research prioritization.

Important conflicts noted throughout this paper have not been recognized as conflicts of interest, even though they fit Thompson’s definition of a primary interest being undermined by a secondary interest, perhaps because they are structural (built into the system). Clearly, more definitional work is required. The current regulatory non-system for reporting and managing individual financial conflicts of interest is light years away from that needed to rebalance academic and commercial standards and norms in order to pay proper attention to social and environmental aspects of health.

Alternatively, in the current political environment, concerns about COI can seem quaint. Cultural fault lines fuel opposition to scientific evidence and certainly to its cultural stature in an era in which corporations supported by government policy control the flow of economic capital across the globe. Since many COIs flow from corporate interests, their power neuters the notion that there is a conflict or one that would do damage. At the same time, research-based regulations suffer less stature because they flow from science-based institutions, whose cultural stature has diminished (84). And the current political and medical environment invites and sustains fake news, mis/disinformation, resulting in the widespread dissemination of misleading and biased information (85).

This manuscript contains limitations which will require additional conceptual and empirical work including experiences of a range of countries and industries. As noted earlier, it is written in the mode of a narrative critical review, aimed at critique and insight. It is also largely US focused. Several next steps are necessary before its premises can be explored by the global research community.

In conclusion, the current operative definition of conflict of interest is almost exclusively focused on individual conflicts (micro level), less so to institutional conflicts of interest (mezzo level). This paper argues that the purposes for controlling conflict of interest cannot be attained until structural conflict of interest (macro level) is operative.

## Author contributions

BR: conceptualization, writing and editing.

## Conflict of interest

The author declares that the research was conducted in the absence of any commercial or financials relationships that could be construed as a potential conflict of interest.

## Publisher’s note

All claims expressed in this article are solely those of the authors and do not necessarily represent those of their affiliated organizations, or those of the publisher, the editors and the reviewers. Any product that may be evaluated in this article, or claim that may be made by its manufacturer, is not guaranteed or endorsed by the publisher.
